# Early and transient reverse transcription during primary deltaretroviral infection of sheep

**DOI:** 10.1186/1742-4690-5-16

**Published:** 2008-02-01

**Authors:** Carole Pomier, Maria T Sanchez Alcaraz, Christophe Debacq, Agnes Lançon, Pierre Kerkhofs, Lucas Willems, Eric Wattel, Franck Mortreux

**Affiliations:** 1 CNRS FRE3011-Université Claude Bernard, Oncovirologie et Biothérapies, Centre Léon Bérard, Lyon, France; 2 FUSAGx, Molecular and cellular biology, Gembloux, Belgium; 3 Hôpital Edouard Herriot, Service d'Hématologie, Pavillon E, Lyon, France; 4 Veterinary and Agrochemical Research Centre, Department of Virology, Uccle, Belgium

## Abstract

**Background:**

Intraindividual genetic variability plays a central role in deltaretrovirus replication and associated leukemogenesis in animals as in humans. To date, the replication of these viruses has only been investigated during the chronic phase of the infection when they mainly spread through the clonal expansion of their host cells, vary through a somatic mutation process without evidence for reverse transcriptase (RT)-associated substitution. Primary infection of a new organism necessary involves allogenic cell infection and thus reverse transcription.

**Results:**

Here we demonstrate that the primary experimental bovine leukemia virus (BLV) infection of sheep displays an early and intense burst of horizontal replicative dissemination of the virus generating frequent RT-associated substitutions that account for 69% of the in vivo BLV genetic variability during the first 8 months of the infection. During this period, evidence has been found of a cell-to-cell passage of a mutated sequence and of a sequence having undergone both RT-associated and somatic mutations. The detection of RT-dependent proviral substitution was restricted to a narrow window encompassing the first 250 days following seroconversion.

**Conclusion:**

In contrast to lentiviruses, deltaretroviruses display two time-dependent mechanisms of genetic variation that parallel their two-step nature of replication *in vivo*. We propose that the early and transient RT-based horizontal replication helps the virus escape the first wave of host immune response whereas somatic-dependent genetic variability during persistent clonal expansion helps infected clones escape the persistent and intense immune pressure that characterizes the chronic phase of deltaretrovirus infection.

## Background

Retroviruses are unique in that they exist as DNA and/or RNA species. Their polymerases are reverse transcriptases devoid of 3' exonucleolytic activity, and genetic variability is thereby a part of their way of life [1]. Among retroviruses, deltaretroviruses possess an additional mechanism of replication that accompanies an original way of genetic variability. In addition to reverse transcriptase, that generate an error rate in the same range as those of other retroviruses; these lymphotropic viruses encode regulatory proteins that interfere with many host cell pathways including cell cycle, apoptosis and DNA repair [2,3]. This results in the persistent clonal expansion of infected cells and generates a significant level of genetic variability resulting from somatic mutations of the proviral sequence [4-6].

Deltaretroviruses include human T-cell leukemia viruses type -1 [7] and -2 (HTLV-1 and 2) [8], the recently discovered HTLV-3 [9] and -4 [10], simian T-cell leukemia viruses (STLV) [11], and the bovine leukemia virus (BLV) [12]. They infect vertebrates and cause leukemia and lymphoma. Two steps characterize the course of deltaretroviruses infection in vivo, including a brief period of primary infection followed by chronic and persistent infection [4,6,13,14]. After experimental infection, primary infection starts with viral contamination and, at least for HTLV-1 in squirrel monkey (*Saïmiri sciureus*) and BLV in sheep, finishes 1–6 months later, as soon as both humoral and cellular antiviral host immune responses have been mounted [6,15]. The second phase of the infection encompasses the remaining lifespan of infected organisms. It can be clinically latent or associated with the development of inflammatory or malignant diseases. The somatic mutation process that governs deltaretroviruses genetic variability in vivo characterizes the chronic phase of the infection, including asymptomatic and disease states. During this period, RT-associated substitutions have never been detected in transformed or untransformed clones [4,5,14,16]. However, the mechanisms underlying deltaretroviruses genetic variability, i.e. somatic versus RT-associated mutations, have not been investigated in vivo during the primary infection. Here we investigated for the first time the genetic variability process of a deltaretrovirus in vivo during primary infection. By monitoring BLV replication during early experimental sheep infection we detected a transient burst of RT-generated mutations.

## Results

### Experimental strategy

**Figure 1:**
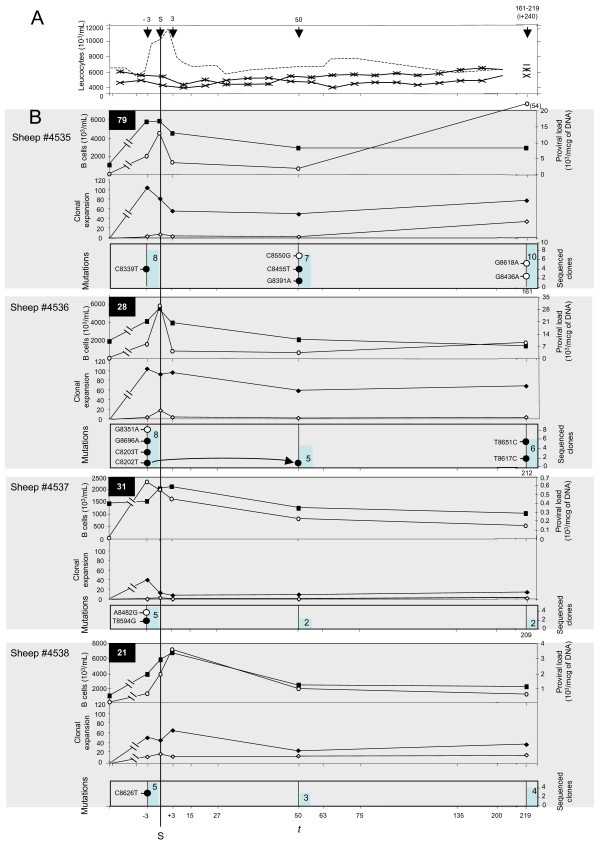
Early bovine leukemia virus replication in experimentally infected sheep. Vertical arrows represent the times at which blood samples were collected. A fluctuation of circulating leukocyte counts over time. ----- mean leukocyte counts of the 4 experimentally BLV-infected sheep aligned relative to the date of seroconversion -x-x-x-x- leukocyte counts of the two uninfected sheep, aligned relative to the date of injection of the non-infectious solution. B BLV early replication in experimentally infected sheep. All curves are aligned relative to the date of seroconversion (S). Time (t) is expressed in days. For each animal the first 2 curves represent the temporal fluctuation of the B cell count (black squares) and proviral loads (open circles); the second 2 curves represent the clonality of BLV positive circulating cells (black rhombuses, clones = 1200 copies in 1 mcg of circulating DNA; white rhombuses, clones >1200 copies in 1 mcg of circulating DNA); bottom curves represent the frequency of RT-associated substitutions (black circles) and of somatic mutations (open circles); the blue bars represent, at each time, the number of sequenced BLV integration sites.

Four sheep were experimentally infected with BLV infectious molecular clones pBLV344 or pBLVIG4. These viruses are known to induce persistent infection in this experimental host. As previously described and shown in Figure 1A, experimental primary BLV infection in sheep resulted in transient hyperleukocytosis whereas no significant fluctuation of circulating leukocyte counts characterized control animals [17,18]. Animals #4535, 4536, 4537, and 4538 seroconverted 79, 28, 31, and 21 days after experimental infection, respectively. For these 4 experimentally infected sheep, B lymphocytosis, circulating proviral loads, and clonality were investigated at different times including the date of seroconversion, 3 days before, and 3 and 50 days after seroconversion, and 240 days after experimental infection (Figure 1B).

#### Early BLV replication in experimentally infected sheep

Figure 1B shows that, for each animal, circulating BLV proviral loads paralleled B cell counts; these two variables were significantly correlated when data from the 4 experimentally infected sheep were pooled for statistical analysis (p < 0.002 and Spearman's rho = 0.39). The quadruplicate inverse PCR amplification of 3' BLV integration sites permitted to estimate both the number of circulating integrated BLV proviruses and their degree of expansion through the clonal expansion of their host cells. For each animal, the most abundant clones, i.e. those detected more than 2 times after quadruplicate IPCR and corresponding to a clonal frequency of >1/1200, were distinguished from those harboring a lower detection frequency (Figure 1B).

**Figure 2:**
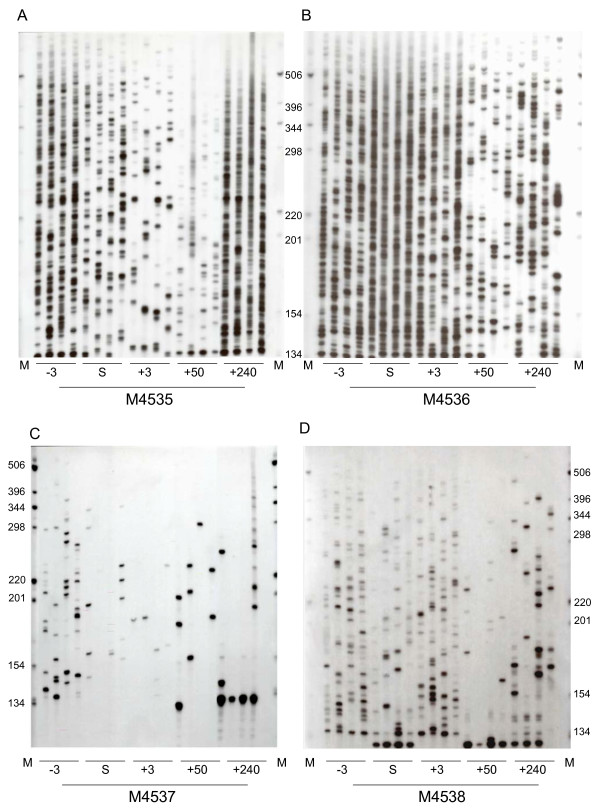
Clonality of BLV-infected cells over time in animals 4535 (A), 4536 (B), 4537 (C), and 4538 (D). Each sample was analyzed in quadruplicate by IPCR as detailed in the Material and Methods section. Each signal on the gel represents a cluster of BLV integration sites having the same length and therefore belonging to the same cellular clone. The absolute detection threshold of the technique was ~21 copies/150,000 PBMCs while samples harboring 1, 2, 3, and 4 signals after quadruplicate experiments corresponded to a BLV clonal frequency of 25 to 62.5, 62.5 to 1200, 1200 to 2400, and > 2400 infected cells per 150,000 PBMCs [4].

Figure 2 represents the temporal fluctuations of the BLV integration pattern for the 4 experimentally infected sheep. The animals displayed roughly parallel clonality patterns (Figure 1B) with an early and transient increase of the number of clones which subsequently decreased to reach a relatively stable level ~50 days after seroconversion. Figure 1B shows that the number of polyclonally expanded clones increased earlier than that of abundant clones, with, for each animal, a 3-day interval between the first 2 peaks. Figures 1B and 2 show that during primary infection a burst of clonal expansion characterized the period of seroconversion. With the exception of animal 4537 for which the zenith of proviral load coincided with that of the overall number of clones, figures 1B and 2 show that the number of circulating BLV proviral copies better correlated with the degree of clonal expansion, i.e. with the number of abundant clones. This correlation was statistically significant when these 2 data (circulating BLV proviral copies and number of abundant clones) were pooled for the 4 animals (p < 10^-4 ^and Spearman's rho = 0.76). In animal 4535, the number of abundant clones increased during the course of the infection and the extensive proliferation of a subset of these clones accounted for a significant increase of the circulating proviral load over time (Figure 1B and 2). At distance from the seroconversion date, the clonality pattern of the remaining 3 animals remained stable over time during the period of the study. These results indicate that BLV primoinfection, i.e. the first months consecutive to the infection of sheep, includes a first burst of both polyclonal distribution and extensive clonal expansion of infected cells, which results in a transient peak of circulating proviral load.

#### RT-versus somatically-generated BLV sequence mutations during early infection in vivo

**Figure 3:**
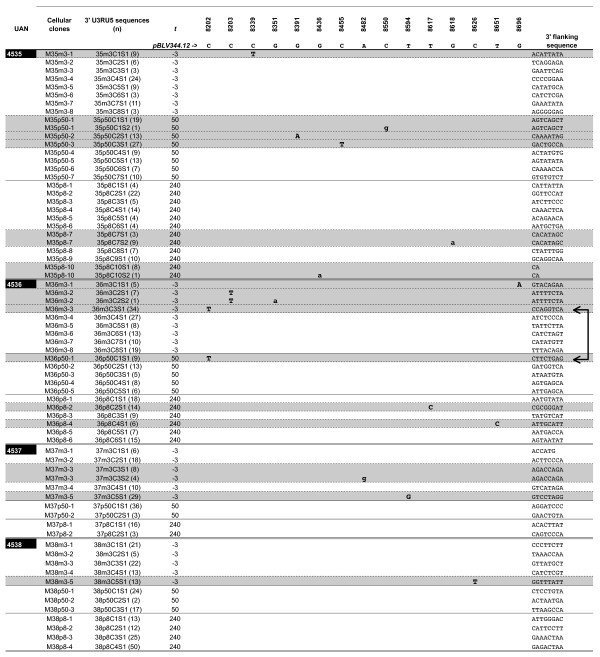
Somatic (miniscule letters) versus reverse-transcriptase (capital letters) associated substitutions of the BLV 3' RU5 sequence during early experimental sheep infection. Overall, 65 distinct BLV 3' integration sites were isolated; the first 8 bases of the corresponding flanking cellular sequences are given on the right. RU5 sequences were aligned according to the sequence of the wild type BLV sequence 344 used for experimental infection of animals 4535 and 4536. Sheep are identified by their unique animal number (UAN). Each cluster of RU5 sequences sharing a common integration site, and therefore belonging to a unique clone of expanded B cells, is identified by its cellular clone number. For each cellular clone the number of non-unique 3' U3RU5 consensus sequences is indicated between brackets in the third column. Cellular clones harboring a mutated 3'U3RU5 sequence are overlined in grey. A horizontal double bar separates the clusters of sequences derived from each of the 4 sheep DNA samples. For each animal, cellular clones are sorted according to their date of isolation, i.e. 3 days before, 50 after seroconversion and 240 days after experimental infection. The two horizontal arrows represent the two times at which the same C8202T substitution was observed in 2 distinct sequences deriving from animal #4536.

**Figure 4:**
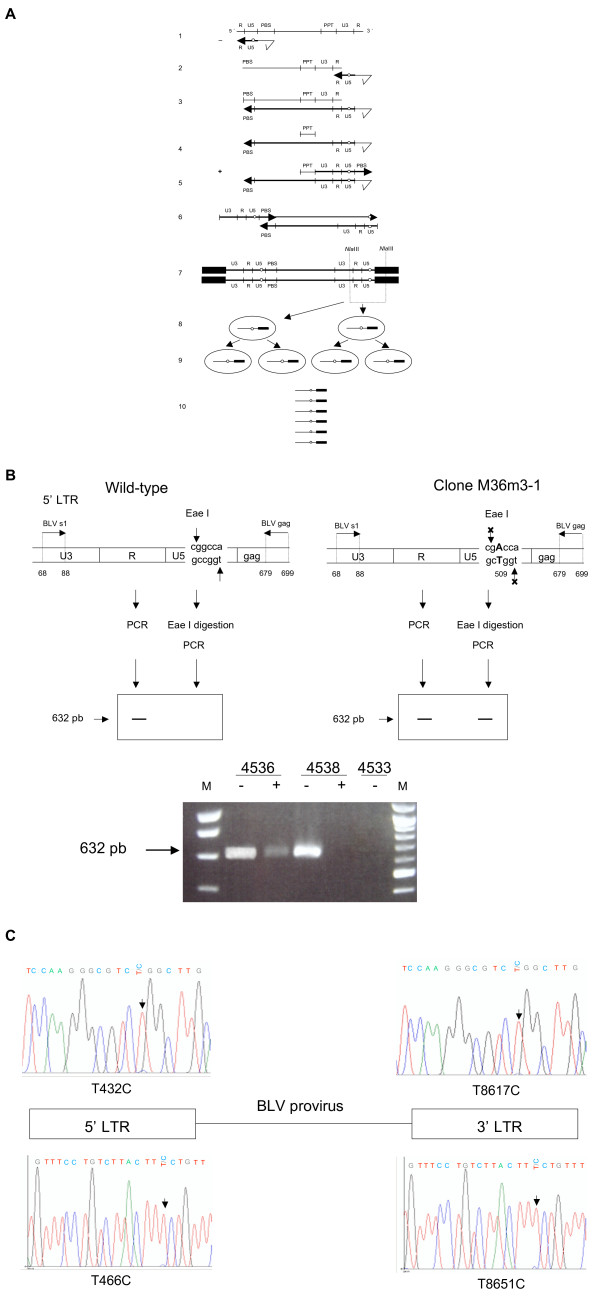
RT-associated mutations frequently occur during BLV minus strand synthesis. **A **Generation of U5 substitution during minus strand synthesis. RNA is represented as a thin line whereas DNA is represented as thick lines. The first 6 horizontal lines represent the synthesis of the provirus. Line 7 represents the integrated provirus flanked with its two integration sites represented as black boxes. NlaIII restriction sites are represented on both the provirus and the 3' cellular flanking sequence. Lines 8 and 9 represent the first two mitoses of the infected cells harboring the integrated provirus. For each cell, the RU5 sequence and the 3' flanking sequence encompassed by the 2 NlaIII restriction sites, i.e., the sequences obtained after inverse PCR, are represented. Line 10 represents the sequences obtained after inverse PCR, cloning and sequencing. The open circle represents a RT-associated mutation that has occurred during the synthesis of the 5' RU5 minus strand. As shown in lines 1 to 6, this substitution appears to be harbored by both strands of the two LTRs of the integrated provirus. Accordingly, all infected cells from the corresponding clone (identified by their common integration site) harbor a provirus with the same substitution (see lines 8 and 9). As a consequence, all sequences from this clone obtained after inverse PCR harbor the same mutation at the same position. **B **PCR detection of the G511A U5 substitution along the 5' LTR. Top: 5' BLV LTR from the wild-type (left) and from clone M36m3-1 (right) carrying the putative G511A substitution having occurred during minus strand synthesis, thereby generating the G8696A substitution identified by sequencing IPCR product derived from the sample harvested in animal 4536 three days before seroconversion. In the absence of digestion, specific 5' LTR PCR amplifies a fragment of 632 bp. EaeI digestion of the wild-type sequence abolishes this signal while incubation with EaeI has no effect on the sequence carrying the G511A mutation, leading to the detection of the 632 bp fragment. Samples studied in the presence (+) or in the absence (-) of EaeI digestion derived from BLV infected animals 4536 and 4538, both harvested 3 days before seroconversion, and from the uninfected control animal 4533. **C **Detection of the T8617C and T8651C substitutions in their corresponding positions along the 5' LTR. Each LTR was specifically amplified by PCR and substitutions were detected by direct sequencing of PCR product, as detailed in the experimental procedures.

We searched for RT-versus somatically-associated substitutions of the BLV provirus by comparing the nucleotide composition of 3'BLV RU5 sequences flanked by distinct versus identical integration sites, as previously described for BLV or HTLV-1 [4,6]. For each experimentally infected animal, IPCR products obtained 3 days before, 50 days after the date of seroconversion, and 240 days after experimental infection were cloned without size selection. A total of 842 molecular clones were sequenced (370 kbp of proviral sequence with 64 kbp of integration site) and could be arranged into 65 distinct cellular clones based on cellular flanking sequences. The number of cellular clones analyzed for the 4 sheep is represented in Figure 1B; at each time and for each animal, it was correlated with the overall number of detected clones (Figure 1B). BLV sequences were aligned with respect to infectious proviral clone BLV-p344, which was taken as a reference (Figure 3). Fifteen of 65 (23%) cellular clones harbored mutated 3' LTR sequences (16 substitutions), the number of mutations per sequence ranging from 0 in 660 sequences, 1 in 181 sequences and 2 in one sequence. The 16 substitutions were distributed as 14 transitions and 2 transversions (Figure 3). For 10 cellular clones (M35m3-1, M35p50-2, M50p50-3, M36m3-1, M36m3-3, M36p8-2, M36p50-1, M36p8-4, M37m3-5, M38-m3-5, Figure 1B, Figure 3 (shaded in light gray), and Figure 4), all the 3'RU5 sequences defining the clones shared a common and clone-specific substitution whereas 4 additional cellular clones included only a subset (1/20 to 9/12) of mutated 3'LTR sequence (dark gray shading, Figure 3). The distribution of the former corresponded to that of RT-associated mutations whereas that of the latter possessed the hallmark of somatically generated mutations [16,19], which are only harbored by a subset of sequences belonging to a given clone. An additional clone isolated from sheep #4536 three days before seroconversion (M36m3-2, Figure 1B and Figure 3) harbored eight 3'RU5 sequences with the same C8203T transition; one of these sequences had an additional G8351A transition. This additional clone therefore harbored a RT-mutated 3'RU5 sequence having subsequently undergone a G8351A somatic substitution. All detected mutations were clearly beyond the level of PCR errors or artifacts, which were estimated for this region to be <1 per 30 kb sequenced [4,16]. For the first time for a deltaretrovirus, these results provide evidence that early BLV replication is RT-dependent, and generates a mutation load accounting for 69% of the provirus genetic variability.

#### RT-associated mutations frequently occur during BLV minus strand synthesis

As shown in Figure 4A, present RT-associated substitutions possessed the hallmarks of minus-strand synthesis-associated mutations [16,19]. Those are typically present on both 3' and 5' LTRs [16,19]. Among these, the G8696A substitution harbored by clone M36m3-1 (sequence 36m3C1S1, Figure 3) and present 3 days before seroconversion in sheep 4536 abolished a restriction site for the Eae I enzyme (YGGCCR->YGACCR where R is a purine and Y a pyrimidine, Figure 4B). We next searched for this G->A substitution along the 5' LTR, i.e. at position 511. Oligonucleotides BLV-s1 and BLV-gag encompassing the Eae I restriction site at position 511 within the 5' RU5 sequence were used for PCR amplification (see experimental procedures). To specifically amplify the 5' LTR rather than its 3' counterpart, we chose a 3' primer, BLV-gag, complementary to the gag gene of the BLV proviral sequence (Figure 4B). In the absence of Eae I digestion, PCR amplification of the BLV provirus with BLV-s1 and BLV-gag primers generated a PCR product of 632 bp (Figure 4B). In the absence of substitution within the Eae I restriction site, PCR amplification of Eae I digested DNA gave no signal whereas, after incubation with Eae I and PCR amplification, G511A mutated sequences could not be digested and thereby generated the 632 bp PCR product (Figure 4B). As shown in Figure 4B, this signal was generated after PCR amplification of the DNA of peripheral blood cells deriving from sheep 4536 on day 3 before seroconversion but not on samples deriving from other infected sheep or from uninfected control. To rule-out the presence of a PCR inhibitor in samples with negative results, a control PCR was performed using a primer set specific for the GAPDH gene, and a specific signal was obtained with all digested DNA samples (not shown). Therefore this control experiment confirmed that the G8696A RT-associated substitutions revealed by cloning 3' IPCR products had occurred during the synthesis of the BLV provirus minus strand in vivo. The T8617C and T8651C substitutions revealed in animal 4536 212 days after seroconversion were harbored by all the sequences belonging to clones M36p8-2 and M36p8-4 respectively (Figures 1B and 3), and were thus assumed to have been generated during RT. Two pairs of primers encompassing the corresponding positions of these substitutions along the 5' (BLV-s1 and BLV-gag) and the 3' LTR (BLV-tax and BLV-U5as) were synthesized (see experimental procedures). After amplification, PCR products corresponding to the sample collected 212 days after seroconversion were directly sequenced and both substitutions were identified along the 3' and 5' LTR. Electropherograms show that 3' and 5' substitutions were harbored by a similar proportion of sequences (Figure 4C). No such signal could be observed when DNA deriving from sheep #4535 or 4538 was assayed. Therefore, as for the G8696A transition, these results suggest that T8617C and T8651C transitions have been RT-generated during minus strand synthesis. Investigating for the first time the period at which substitutions occur during BLV reverse transcription in vivo, these results suggest that the synthesis of the minus provirus strand is more error prone than that of the plus strand.

#### In vivo cell-to-cell passage of a BLV proviral sequence harboring a RT-dependent mutation

The C8202T RT-associated substitution harbored by clone M36m3-3 and isolated from sheep #4536 three days before seroconversion, i.e. 25 days after experimental infection (Figure 1B and 3) was subsequently identified in clone M36p50-1, characterized by a distinct flanking sequence and isolated 53 days later from the same animal. This suggests that clonally expanded cells from clone M36p50-1 shared a RU5 sequence having necessary undergone at least two rounds of horizontal replication. Alternatively but less probably, the two C8202T substitutions might have occurred during two distinct RT cycles.

#### RT-associated substitutions are restricted to early experimental BLV infection

**Figure 5:**
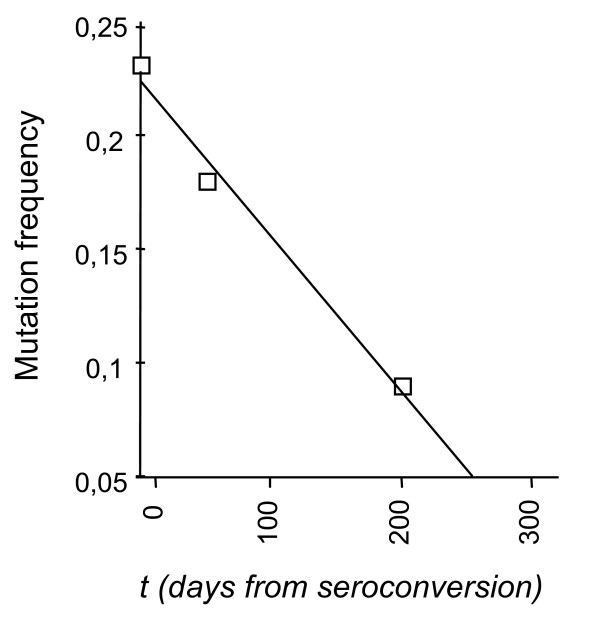
Time-dependent decrease of RT-generated BLV proviral substitutions during early experimental infection. Data from Figure 1 served to plot the frequency of clones harboring RT-dependent substitutions against time in the 4 experimentally infected sheep.

We next investigated the temporal distribution of somatically-versus RT-generated BLV proviral substitutions. The proportion of circulating cellular clones harboring somatically mutated BLV proviral sequences at 3 days before seroconversion, 50 days after seroconversion and 240 days after infection were 7.6%, 5.8%, and 9.5%, respectively. As previously observed with HTLV-1 [16] or BLV [4], this distribution was time-independent. In contrast, during primary BLV infection, the proportion of clones harboring RT-associated mutations was inversely and linearly correlated with time (r^2 ^= 0.99) (Figures 1 and 5). As a consequence, it could be extrapolated from Figure 5 that clones with more than 21 infected cells (the lower limit of IPCR detection) harboring RT-generated mutations could not been detected in the blood flow of experimentally infected animals after the 250th day following seroconversion. These results highlight the ephemeral nature of RT-generated BLV substitutions in vivo.

## Discussion

Deltaretroviruses possess two modes of replication that include the classical horizontal retrovirus-like spread and the cell-associated clonal expansion of proviral sequences [14,20]. The former generates RT-associated substitutions of the provirus whereas the latter is associated with somatic mutations of both the provirus and the host cell sequence [4,16].

Our work was performed in the sheep experimental model for BLV infection, which is a practical way to study early infection in vivo. Events occurring in the present model after injection of proviral DNA may not reflect what occurs after natural transmission in cows, namely cell-associated infection followed by horizontal and vertical virus spreads. However, we have demonstrated ([4] and present results) that the inoculation of infectious molecular BLV clones in sheep triggered a temporal pattern of infection similar to that observed after experimental cell-associated infection [6], i.e. the generation of newly infected host-cells followed by their persistent clonal expansion. Together these data contribute to validate the present experimental model at the replicative level.

BLV infection of sheep regularly triggers the formation of tumors that which occur faster than in the small percentage of infected cattle that develop tumors [21-25]. It is possible that events occurring during early infection in the sheep model may set the stage for rapid tumor development. Alternatively, one can propose that the better adaptation of the virus to its natural host might contribute to the significantly lower incidence of BLV-related malignancies in cattle.

The experimental strategy used in our study permitted to monitor in vivo these two routes of BLV replication and genetic variability over time. This allowed to show that experimental primary BLV infection of sheep includes an early and intense burst of both horizontal and vertical viral disseminations, generating frequent RT-associated proviral substitutions that account for 69% of the in vivo BLV genetic variability during the first months of the infection (Figure 1, 2, 3). However, all 4 experimentally infected sheep displayed a rapid shift towards a predominant vertical route of replication as demonstrated by the fact that no RT-dependent substitution could be detected from the 250th day after seroconversion.

Present results about BLV genetic variability are the first evidence of a RT-dependent mutation process for a deltaretrovirus in vivo. Typically, both RNA-dependent and DNA-dependent DNA syntheses by RT contribute to the genetic variability of retroviruses. In addition, RNA transcription by cellular RNA polymerase II could also participate to the mutation process. However the error rate of RNA polymerase II and its contribution to retroviral mutation rates remain unknown. In vitro studies of mutation rates during RNA- and DNA-dependent HIV DNA synthesis have produced conflicting results. They suggest a higher mutation rate during RNA-dependent DNA synthesis [26], a higher mutation rate during DNA-dependent DNA synthesis [27], or equal mutation rates during RNA- and DNA-dependent DNA syntheses [28]. In addition, it appears from in vivo studies that some elements affecting fidelity in vivo are absent in *in vitro *assays [29-33]. In vivo mutation rates have been measured for the bovine leukemia virus [34] however the contributions of the various nucleic acid polymerization steps in retroviral replication to the in vivo retroviral mutation rates have not been evaluated. From the present experimental study, in vivo RT-dependent mutations appeared to mainly occur during BLV minus strand synthesis.

All 4 experimentally infected sheep displayed an early burst of horizontal BLV replication that generated a burst of RT-dependent proviral substitutions (Figure 1). In contrast, the transient peak of clonal expansion that accompanied the intense horizontal spread was not found to increase the detection frequency of somatic mutations (Figures 1 and 2). Previous studies have clearly linked the degree of clonal expansion with the somatic mutation frequency [4,14,16]. During natural HTLV-1 infection, heavily expanded clones regularly display the highest somatic mutation frequencies, which culminate at the malignant stage [6]. Similarly, in experimentally infected sheep, the premalignant and malignant BLV positive clones harbor the highest degree of clonal expansion together with the highest somatic mutation loads, when compared with other clones of infected cells [4]. Therefore the present loss of correlation between clonal expansion and somatic mutations seems to be at odds with these previous results. However, those were obtained by investigating the chronic phase of the infection in organisms having mounted the specific and robust adaptive antiviral immune response characteristic of deltaretrovirus infection [4,16]. In the present study, the absence of such a strong immune response that characterizes early infection might account for the absence of detected somatic mutations at this stage of the infection. In other words, together with previous findings, present results are consistent with the idea that the host immune response might be involved in the selection of somatic mutations, thus explaining why the correlation of their frequency with the degree of clonal expansion is restricted to the chronic phase of the infection.

The detection of RT-associated proviral substitutions was confined to a narrow window encompassing the first 250 days following seroconversion. This is in agreement with our previous works on HTLV-1 [16] and BLV [4], which regularly failed to identify RT-associated proviral mutations in circulating, infected clones in vivo during the late phase of the infection [4,14,16]. The question remains of how these RT-acquired BLV substitutions disappear over time. The time-dependent decrease in their detection frequency (Figure 5) parallels the time-dependent development of the robust and subsequently persistent anti-BLV immune response [35,36]. Together with present results, this suggests that RT-generated substitutions and viral expression could be synonymous. Accordingly, after integration, RT-dependent mutated proviral sequences undergo a negative immunological control for clonal expansion. This hypothesis also helps explain why newly generated RT-dependent substitutions have never been detected during the chronic phase of deltaretroviruses infection in sheep or in humans [4,16]. Alternatively, RT-dependent substitutions might represent proviruses having undergone modification of key genes involved in the control of host cell multiplication. Finally, given a BLV IPCR detection threshold of 21 copies per microgram of DNA [4], present results do not preclude that weakly expanded sequences harboring RT-dependent substitutions could be generated and/or maintained over time after experimental BLV infection.

In conclusion, our study suggests that, in contrast to other retroviruses, deltaretroviruses possess two time-dependent pathways of genetic variation that parallel their two-step nature of replication over time [6] and correspond to RT-associated rearrangements and somatic mutations. The former appears restricted to the first months of the infection while the latter dominates the prolonged steady-state step of the infection, with the suggestion that this time-dependent pattern of replication depends on the host immune pressure.

## Methods

### Experimental BLV infection of sheep

Six one-year-old sheep were kept under controlled conditions at the Veterinary and Agrochemical Research Centre (Machelen, Belgium). Handling of animals and experimental procedures were approved by the ethics committee and were conducted in accordance with institutional and national guidelines for animal care and use. Four sheep were experimentally infected with BLV infectious molecular clones as previously described [17]. Briefly, 100 μg of circular plasmid DNA was mixed with 200 μg of Dotap (Roche Diagnostics) and injected intradermally into the back of the sheep. Two animals, # 4535 and 4536, were experimentally infected with a BLV wild-type cloned provirus (pBLV344) [37]. A plasmid containing the mutant provirus pBLVIG4, which harbors a stop codon in the G4 open reading frame [17], was injected in sheep # 4537 and 4538. Two additional animals, # 4533 and 4534, received a non-infectious Dotap solution and served as uninfected controls. Twice a week, the total leukocyte counts were determined by using a Coulter counter ZN, and the number of lymphocytes was estimated after examination under the microscope after staining with May-Grunwald Giemsa. In parallel, the sera from each sheep were analyzed for BLV seropositivity using immunodiffusion and enzyme-linked immunosorbent assay (ELISA) techniques [38].

### Immunophenotyping of circulating cells

Peripheral blood mononuclear cells (PBMCs) were isolated by Percoll gradient centrifugation and their viability was estimated by trypan blue dye exclusion [39]. PBMCs were labeled with monoclonal antibodies (Mabs) directed against surface immunoglobulin M (anti-sIgMs, clone 1H4, mouse IgG1; Pig45A2, mouse IgG2b), CD4 (ST4, mouse IgG1), CD8 (CC58, mouse IgG1) provided by C. Howard (Institute for Animal Health, Compton, United Kingdom) and by I. Schwartz-Cornil (INRA, Jouy-en-Josas, France). Cells were then labeled with a rat anti-mouse IgG1 phycoerythrin (PE)-antibody (Becton Dickinson Immunocytometry Systems) or with a goat anti-mouse IgG2b fluorescein isothiocyanate (FITC)-conjugate (Caltag Laboratories). Finally, PBMCs were analyzed by flow cytometry on a Becton Dickinson FACScan flow cytometer. Ten thousand events were collected for each sample and data were analyzed with the Cellquest software (Becton Dickinson Immunocytometry Systems).

### Measurement of circulating BLV proviral Load

The circulating amounts BLV proviral sequences were measured by LightCycler quantitative PCR as described [4]. Briefly, the reaction mixture included polymerase (LightCycler Kit Fast Start DNA Master Hybridization Probes; Roche), 2 mM MgCl 2, 500 nM primer BLVQF, 500 nM primer BLVQR both targeting Px region and 100 nM donor probe 3' end labeled with fluorescein and 200 nM acceptor probe 5' end labeled with LC Red640. Standardization of the amount of DNA subjected to quantification was performed with quantitation of the sheep beta-globin gene as an internal standard [40]. The standard curve for beta-globin was generated using DNA extracted from BLV negative sheep blood cells.

### Detection and quantification of the clonal distribution of circulating BLV positive cells in vivo

BLV integration was analyzed by Inverse Polymerase Chain Reaction (IPCR) as described [4]. Briefly, two micrograms of DNA were digested by 20 U NlaIII and 20 units of MfeI in 1X NlaIII-MfeI buffer for 3 h at 37°C. MfeI digestion was performed in order to avoid the amplification of a 536 bp segment of the 5' LTR complementary to the set of 3' IPCR primers. Digestion was controlled by 1% agarose gel electrophoresis and DNA was extracted with phenol/chloroform (1:1) and precipitated with 100% ethanol. One microgram of digested DNA was circularized for 16 h at 16°C with 20 U of T4 DNA ligase. As there is a stochastic component to the detection of retrovirus integration sites using inverse PCR [41], samples were analyzed in quadruplicate, as previously described for BLV and HTLV-1 [4,41]: 4 × 500 ng of circularized DNA were amplified for 39 cycles using 200 μM of the primer pair BLV3'S and BLV3'AS. Amplifications were performed using 3.5 units of the Pfu DNA polymerase with thermal cycling parameters as follows: 95°C 10 min, 35 × (95°C 1 min, 60°C 1 min, 72°C 3 min), and a final elongation step of 10 min at 72°C. The length polymorphism analysis of 3' BLV flanking sequences was performed by making a run-off. This method consists in the linear PCR amplification of the provirus 3' extremities together with their flanking sequences. Two microliters of amplified IPCR products were submitted to 10 cycles of linear PCR with 2 μM of 5'-32P-radiolabeled primer BLV3'RO. Run-off products were analyzed on 6% sequencing gel. As previously described [4], the stochastic nature of BLV IPCR was found to appear at BLV integration site frequencies ranging between 25 and 2400 copies of the BLV provirus per mcg of blood DNA. At copy numbers ranging from 1200 to 2400, 62.5 to 1200, and 25 to 62.5, detection was 3/4, 2/4, and 1/4, respectively. Accordingly, DNA samples from BLV infected animals were analyzed in quadruplicate (4 × 0.5 mcg).

### Assessment of BLV genetic variability in vivo

The cloning and sequencing of 3'LTR-integration site PCR fragments were performed as previously described [4]. Briefly, purified IPCR products were ligated with SmaI-digested and M13mp18 replicative form DNA. After transformation of Escherichia coli XL1, recombinant M13 plaques were screened by hybridization with the BLV3'RO or the BLV5'RO LTR-specific 32P-labelled oligonucleotides. Single-stranded templates were sequenced using fluorescent dideoxynucleotides. The sequenced products were resolved on an Applied Biosystems 377A DNA sequencer with 377A software. Sequence alignments were performed with Sequence Navigator Software.

### Detection of proviral mutations by direct sequencing of PCR products

Specific 3' versus 5' LTR oligonucleotides were used for PCR, and PCR products were directly sequenced. PCR amplifications of 5'- and 3'-LTRs were performed with oligonucleotides encompassing the 5'-RU5 sequence (BLV-s1 5'-AGAAAAGCTGGTGACGGCAG-3' and BLV-gag 5'-GCTTTGCAGAAGGTTGAGCC-3') and the 3' counterpart (BLV-tax 5'-ACCTGGTCCGAATTGGTTGC-3' and BLV-U5as 5'-GTTTGCCGGTCTCTCCTG-3') respectively. For the amplification of the GAPDH gene, the primers G3PDHS 5'-GACCCCTTCATTGACCTCAACTACA-3' and G3PDHAS 5'-CTAAGCAGTTGGTGGTGCAG-3' permitted to rule-out the presence of PCR inhibitor in DNA sample. Overall, DNA was amplified using 3.5 units of the Pfu DNA polymerase with thermal cycling parameters as follows: 95°C 10 min, 35 × (95°C 1 min, 58°C 1 min, 72°C 3 min), and a final elongation step of 10 min at 72°C. PCR amplified fragments were separated on a 1% agarose gel and visualized by ethidium bromide staining. PCR products were purified using a MinElute PCR Purification kit (QIAGEN, Valencia, CA), and directly sequenced with BigDyeTM Terminator Cycle Sequencing v2.0 Ready Reaction Kit (Applied Biosystems, Foster City, CA) according to the manufacturer's instructions. All PCR products were sequenced directly in both directions with an internal oligonucleotide BLV-2S 5'-CTTCCCCTTTCCCGAAAAAT-3' and the BLV-gag and BLV-U5as for LTR5' and LTR3' respectively. To rule out incorporation errors by Taq polymerase, direct sequencing was repeated from a new amplification reaction. Sequenced products were resolved on an Applied Biosystems 377A DNA sequencer as described above.

### Analysis of the 5' LTR restriction fragment length polymorphism by PCR amplification of digested DNA

The presence of a G511A transition was checked along the 5'LTR. As this G511A substitution abolishes an Eae-I restriction site, DNA (500 ng) was digested by 1 U of Eae-I enzyme in 1X Eae-I buffer for 2 h at 37°C. Digested DNA was subsequently PCR amplified with the BLV-s1 and BLV-gag 5' LTR specific primers. The presence of the substitution was evidenced by gel electrophoresis.

### Control PCR

To check the accuracy of the IPCR and the absence of PCR-associated recombination, 3 cloned 3' BLV U3RU5 sequences flanked by there integration sites and harboring distinct mutations were used as controls. Two hundred and fifty copies of each of these 3 cloned sequences were mixed in 1 μg of uninfected DNA. Five hundred nanograms of mixed DNA were amplified for 35 cycles using 200 μM of BLV3'S and BLV3'AS primer pair under the same conditions as used in the analysis of DNA samples from sheep. Purified PCR products were cloned and sequenced as described above. Fifty-two sequences were obtained and analyzed by CLUSTAL alignment with Sequence Navigator Software.

### Statistical analysis

SPSS statistical software version 11 and CA-Cricket Graph III were used for analyses. The correlation of data was assessed by Spearman's *Rho *nonparametric method. P < 0.05 was considered significant in all analyses.

## Competing interests

The author(s) declare that they have no competing interests.

## Authors' contributions

CP carried out the most experimental work. MTSA, CD and FM performed the sample collections. AL, CP and FM performed the sequencing of IPCR products and the determination of the proviral loads. PK and LW were responsible for the sheep studies and participated to interpretation of data. FM and EW were responsible for the design of the study and its coordination. CP, EW, and FM wrote the manuscript. All authors read and approved the final manuscript.
